# Apitherapy for Parkinson's Disease: A Focus on the Effects of Propolis and Royal Jelly

**DOI:** 10.1155/2020/1727142

**Published:** 2020-10-17

**Authors:** Amira Mohammed Ali, Hiroshi Kunugi

**Affiliations:** ^1^Department of Mental Disorder Research, National Institute of Neuroscience, National Center of Neurology and Psychiatry, Tokyo, Japan; ^2^Department of Psychiatric Nursing and Mental Health, Faculty of Nursing, Alexandria University, Alexandria, Egypt; ^3^Department of Psychiatry, Teikyo University School of Medicine, Tokyo, Japan

## Abstract

The vast increase of world's aging populations is associated with increased risk of age-related neurodegenerative diseases such as Parkinson's disease (PD). PD is a widespread disorder characterized by progressive loss of dopaminergic neurons in the substantia nigra, which encompasses a wide range of debilitating motor, emotional, cognitive, and physical symptoms. PD threatens the quality of life of millions of patients and their families. Additionally, public welfare and healthcare systems are burdened with its high cost of care. Available treatments provide only a symptomatic relief and produce a trail of noxious side effects, which increase noncompliance. Hence, researchers have recently focused on the use of nutraceuticals as safe adjunctive treatments of PD to limit its progress and associated damages in affected groups. Propolis is a common product of the beehive, which possesses a large number of therapeutic properties. Royal jelly (RJ) is a bee product that is fed to bee queens during their entire life, and it contributes to their high physical fitness, fertility, and long lifespan. Evidence suggests that propolis and RJ can promote health by preventing the occurrence of age-related debilitating diseases. Therefore, they have been used to treat various serious disorders such as diabetes mellitus, cardiovascular diseases, and cancer. Some evolving studies used these bee products to treat PD in animal models. However, a clear understanding of the collective effect of propolis and RJ as well as their mechanism of action in PD is lacking. This review evaluates the available literature for the effects of propolis and RJ on PD. Whenever possible, it elaborates on the underlying mechanisms through which they function in this disorder and offers insights for fruitful use of bee products in future clinical trials.

## 1. Introduction

The numbers of aged population are exponentially expanding worldwide. Statistics predict that by 2050 the number of older adults over the age of 80 will grow from 11.5% to almost the double (21.0%) [1, 2]. Aging is associated with progressive functional decline, which results from chronically increased production of free radicals and inflammatory mediators, which in turn promote long-term alterations in cellular structure and function. Numerous physiological alterations contribute to cellular senescence such as mitochondrial dysfunction, telomere shortening, gene methylation, epigenetic alterations, protein misfolding and proteotoxicity, and distorted nutrient sensing [3]. Thus, increased human survival comes at the cost of heightened vulnerability to various age-related pathologies.

Parkinson's disease (PD) is an age-related neurological disorder that affects more than 6.3 million people [4, 5] accounting for around 2% of the world's population [6], which makes it the second most prevalent neurodegenerative disease worldwide [7]. The key pathological hallmark of the disease is chronic, progressive, and selective degeneration of dopaminergic neurons in the substantia nigra pars compacta (SNC) that results from intraneuronal accumulation of misfolded proteins, mainly the synaptic protein *α*-synuclein, a major component of Lewy bodies [4, 6, 8].

## 2. The Pathology Underlying Parkinson's Disease

PD is a multidimensional condition that results from various factors: heredity, lifestyle, nutritional, hormonal, physical, and psychosocial. Evolving knowledge indicates that PD pathology first occurs in the olfactory mucosa and the gut in response to alterations of gut microbiota as a result of direct exposure to ingested toxins or pathogens [6, 9]. *α*-Synuclein—a major contributor to neuronal death in PD—appears in enteric nerves and enteroendocrine cells of PD patients, which is associated with the occurrence of gastrointestinal (GI) symptoms in 70–80% of patients as early as a decade before motor and neurological signs appear. Experimentally, misfolded *α*-synuclein appears in enteric nerves before it appears in the brain while injecting the intestinal wall with abnormal *α*-synuclein leads to its spread into the vagus nerve. Given that vagotomy is associated with a low risk of PD development, the vagus nerve represents the main anatomically interconnected network through which initial seeds of *α*-synuclein get physically transmitted from the gut to neurons in the midbrain. Uptake of *α*-synuclein by vulnerable neurons induces pathological misfolding of *α*-synuclein in recipient neurons, which is associated with further transmission to neighboring cells leading to sporadic spread of PD pathology and further progression of the disease [9–11].

The activity of *α*-synuclein in PD brains is further exacerbated by oxidative stress, a principal contributor to neurodegeneration in PD. PD brains exhibit exceptionally high levels of autoxidation and enzymatic oxidation of dopamine resulting in high emission of reactive oxygen species (ROS). ROS stimulates mitochondrial dysfunction of respiratory chain complex I in the SNC resulting in activation of apoptotic signaling pathways, finally leading to neuronal cell death [12, 13]. In addition, mitochondrial impairment—caused by mutations in certain genes such as *α*-synuclein, parkin, protein deglycase (DJ-1), and PTEN-induced kinase 1 (PINK1)—leads to further ROS production in dopaminergic neurons of the SNC resulting in several brain changes such as higher metabolic stress, increased synaptic aberrations, and low expression of neuroprotective factors, which all lead to a highly selective nigrostriatal dopaminergic degeneration [12, 14–16].

ROS activate matrix metalloproteinases (MMPs), a group of enzymes that are produced in an inert form and get activated by free radicals, hypoxia, infection, inflammation/immunological reactions, and enzymes that free the cysteine bond or cleave the propeptide region. Once activated, MMPs attack the extracellular matrix, basal lamina, and tight junctions in endothelial cells of the blood-brain barrier resulting in increased permeability/leakage, vasogenic edema, increased extracellular space, hemorrhagic transformations, and acute neuroinflammation [17]. Stromelysin-1 (MMP3) is the main MMPs involved in PD pathogenesis. Apoptotic dopaminergic neurons produce large amounts of active MMP3, ROS, and inflammatory mediators. These molecules induce mutations in genes involved in lipid metabolism in the brain such as iPLA2-VIA resulting in dysregulations in the metabolism of fatty acids through a mechanism that involves shortening of acyl-chain in phospholipids, which interferes with mitochondrial functioning, homeostasis, structure of synaptic vesicles, and neurotransmission in the endoplasmic reticulum resulting in high loss of dopaminergic neurons. In addition, depletion of iPLA2-VIA is associated with loss of *α*-synuclein affinity for phospholipids with shorter fatty acyl chains evoking increases in *α*-synuclein accumulation, neuroinflammation, migration of reactive microglia into the pathological region, barrier leakage, and reduction of immune cells infiltration into the SNC leading to increased *α*-synuclein accumulation in neurons and further apoptosis and cell death due to activation of apoptotic signaling cascade involving c-Jun N-terminal kinases and its downstream effector, caspase-3 [4, 18].

Progressive deterioration of motor functions such as resting tremor, rigidity, and bradykinesia is a key feature of PD, primarily associated with dopaminergic neurodegeneration. The Hoehn and Yahr scale is widely used to assess PD patients according to five disease stages, which range from only unilateral involvement in stage I to being wheelchair- or bed-bound in stage V [19]. Nonetheless, the clinical picture of the disease varies considerably among patients, even within the same stage. This is because PD encompasses other major nondopaminergic alterations. Cumulative evidence denotes that degeneration of dopaminergic neurons in the SNC of patients with PD occurs mainly at stage III, along with a concomitant loss of serotonergic neurons in the serotonergic raphe nuclei and cholinergic neurons in the nucleus basalis of Meynert and in the perineuronal nets [20, 21]. Thereby, PD entails a significant reduction of serotonergic and cholinergic markers, which is associated with decline of cognitive functions, mood dysregulations, fatigue, insomnia, weight loss, autonomic dysfunction, olfactory abnormalities, and GI dysfunction such as constipation, nausea, bloating, drooling, delayed gastric emptying, and prolonged intestinal transit time [10, 12, 19, 21, 22].

**Figure 1:**
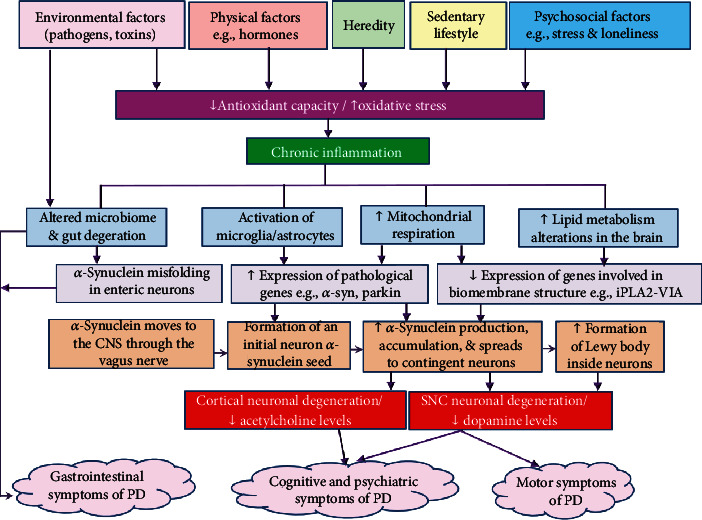
Schematic summary of events contributing to the development of Parkinson's disease. Multiple factors contribute to increased production of free radicals in age people. Meanwhile, the antioxidant capacity decreases with aging, which is associated with chronic increase of inflammatory markers [12, 14, 23, 24]. Inflammation along with free radicals induces morphological and functional mitochondrial alterations resulting in impaired energy production and more emission of free radicals. Injuries of the gastrointestinal tract caused by pathogens and ingested toxins stimulate the expression of the synaptic protein *α*-synuclein in enteric neurons. *α*-Synuclein then moves through the vagus nerve to be seeded in vulnerable neurons in the CNS [9–11]. In the meantime, the expression of genes involved in the synthesis of phospholipids of the biomembrane such as iPLA2-VIA decreases whereas microglia and astrocytes get activated and migrate in response to inflammation and auto-oxidation of dopamine, which trigger the expression of pathological genes such as *α*-synuclein and parkin [18]. As a result, *α*-synuclein pathology increases causing a widespread of initial seeds of *α*-synuclein to the vulnerable neighboring neurons. Consequently, continuous accumulation of *α*-synuclein results in the growth of intracellular tangles to form Lewy bodies inside dopaminergic neurons of the SNC contributing to neuronal dysfunction and death. *α*-Synuclein pathology moves from the SNC into the other brain regions such as the cortex leading to reduction of serotonergic and cholinergic markers such as serotonin and acetylcholine [11, 25]. Accordingly, PD patients undergo serious motor impairments, which decrease gait speed and increase the risk of fall, in addition to a range of other debilitating cognitive, psychiatric, and gastrointestinal symptoms such as poor cognitive performance, mood dysregulation, depression, sleep disturbance, nausea, and chronic constipation—which altogether lower quality of life and increase disability and mortality [20, 24]. ↑ denotes increase; ↓ denotes decrease; CNS: central nervous system; SNC: substantia nigra pars compacta; PD: Parkinson's disease.

The available PD treatment is limited to a single drug, which functions by increasing dopamine levels in the brain to compensate for dopaminergic cell loss. It provides a symptomatic relief of motor symptoms during early stages of the disease [10]. However, it turns to be less effective over time, and it exerts no effect on all nonmotor symptoms. Even more, some of them (e.g., GI symptoms) interfere with the action of the drug and severely alter patients' quality of life [6, 10]. Therefore, recent researches have focused on the use of multifunctional, natural agents, and nutritional modifications as safe adjunctive treatments to impede disease progress, alleviate its symptoms, and promote quality of life in PD patients [6, 19].  Figure 1 briefly summarizes factors associated with PD and its underlying mechanism.

## 3. Apitherapy as a Possible Complementary Treatment for Parkinson's Disease

The beehive produces a large number of products that are loaded with numerous bioactive ingredients such as propolis, bee pollen, honey, royal jelly (RJ), and bee venom [26]. Given the high nutrient content of most bee products, the last few decades have witnessed excessive use of bee products (e.g., RJ, bee pollen, and propolis) as dietary supplements in many parts of the world, especially in Japan [27–30]. In addition, bee products have been historically used as medicines in ancient Egypt and China, and they are gaining research interest as drug targets nowadays [31–35]. Apitherapy is a type of complementary medicine that involves the use of various bee products as therapeutic agents to prevent illnesses and modify disease progression [33, 35].

Research documents antiaging effects of several bee products including RJ and propolis [36, 37]. Accordingly, this review is aimed at investigating the effect of bee products on PD. To retrieve relevant studies, we searched PubMed and Google scholar using a combination of terminologies of “Parkinson's disease” with “royal jelly, honey, bee pollen, propolis, bee venom, bee bread, bee wax, chrysin, apamin, caffeic acid phenethyl ester.” Manual hand-search was also conducted by checking reference lists of the obtained studies. Literature search yielded a number of studies, which involved the use of RJ, bee venom, and propolis to target PD pathology. Given that an existing review has previously evaluated the effect of bee venom on PD [7], the current review focused on examining the effect of other bee products (propolis and RJ) with further elaboration on their underlying molecular mechanism. This section describes active compounds in and biological properties of propolis and RJ in detail.

### 3.1. Composition and Biological Activities of Propolis

Propolis is a malleable, compact, resinous substance that foraging bees form by mixing resin, which they collect from different plant tissues, with bee saliva, wax, and pollen [26]. Its pharmacological properties and color (e.g., green, red, brown, or black) vary according to its botanical source [38]. The word propolis has two Greek parts “pro” and “polis,” which mean “defense” and “city or community.” This name is derived from the function that this substance serves: repairing and sanitizing purposes in bee hives such as sealing holes and cracks, smoothing the inner surface, and maintaining temperature of the hive (e.g., narrowing the hive entrance during cold weather), as well as mummifying and preventing decay of dead pests (e.g., mice) that get into the hives [26, 31].

**Figure 2:**
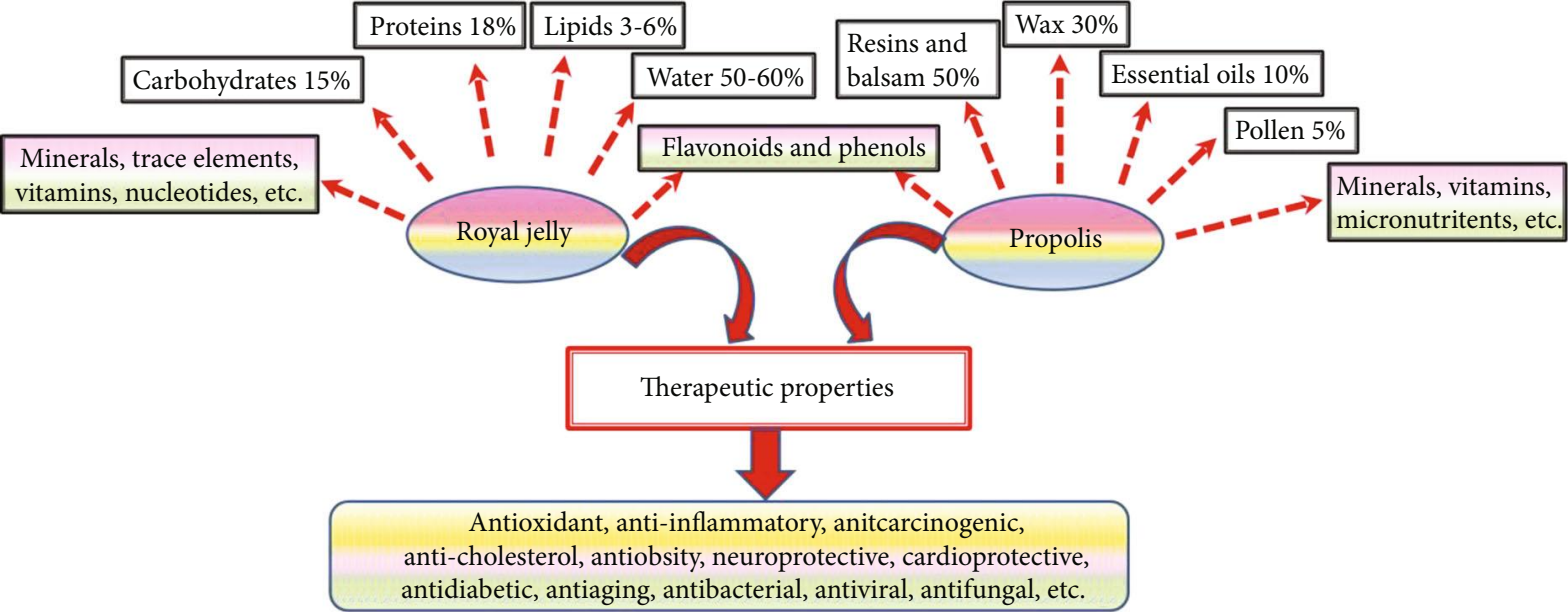
Components of propolis and royal jelly along with their pharmacological activities. Propolis and royal jelly are rich in numerous bioactive elements. Therefore, they express a range of beneficial activities. The key action through which these bee products promote health span stems mainly from their phenolic and flavonoid fractions, which contribute to their strong antioxidant activities. Scavenging free radicals (which activate destructive molecules such matrix metalloproteinases) and enhancing the expression of antioxidant genes promote the suppression of inflammatory responses, thus protecting against cancer, obesity, diabetes, heart disease, neurodegeneration and neurotoxicity, rheumatoid arthritis, and the like.

Propolis has an unusual chemical structure (Figure 2) that involves more than 300 natural compounds [26, 28, 39]. Its content of resins and vegetable balsam, wax, pollen, aromatic, and essential oils, in order, is 50%, 30%, 5%, and 10% [31, 40]. In addition, 5% of propolis comprises different levels of vitamins (thiamin, riboflavin, pyridoxine, niacin, C, and E), amino acids, micronutrients, flavonoids and phenols, phenolic aldehydes, and terpenoids [26, 28, 31, 39, 40]. The limited solubility of crude propolis in water necessitates the use of a suitable solvent (e.g., ethanol, which is the best solvent of propolis) in order to obtain extracts rich in bioactive compounds [40, 41].

Some of the most therapeutic elements in propolis include caffeic acid phenethyl ester (CAPE), chrysin (5,7-dihydroxyflavone), and pinocembrin (PB). All these substances are flavonoids that exist in multiple plants, and they possess strong free radical scavenging potential, on top of many other biological activities, e.g., immunomodulatory, anti-inflammatory, antiviral, and ant-neoplastic [42–45]. PB easily crosses the bloodbrain barrier and produces neuroprotective, antioxidant, and anti-inflammatory activities, which allowed its approval by China Food and Drug Administration as a treatment for ischemic stroke [46].

Due to its total phenolic content (up to 300.36 mg of gallic acid equivalents (GAE)/g of dry weight) and flavonoid content (up to 70 mg of quercetin equivalents (QE)/g), propolis demonstrates a free radical-scavenging activity of about 20 to 190 *μ*g/mL [31, 38]. In general, the exceptional antioxidant effect of propolis is as strong as that of butylated hydroxytoluene, a synthetic antioxidant. However, the antioxidant capacity of various types of propolis varies considerably depending on the type of plant exudates used for their production [31]. Because of its rich chemical composition, propolis has a wide range of several other therapeutic activities: antibacterial, antiviral, antifungal, anti-inflammatory, and anticarcinogenic anticholesterol properties [29, 36, 38, 41, 47, 48]. Accordingly, propolis and its components have been used to promote wound healing and to treat several diseased conditions such as pulp problems, oral candidiasis, genital herpes, and ischemic stroke, to name a few [38, 39, 46, 48].

Propolis is also widely used as a preservative in several food products such as processed meat and wines [40]. Despite the fact that daily consumption of 1.4 mg/kg body weight/day of propolis is relatively safe for humans, individuals with atopy, bee venom allergy, and bronchial asthma can develop a wide range of allergic reactions to this bee product. Hence, individuals with known allergies are advised to completely avoid the consumption of propolis and propolis containing products [49]. Given the enormous benefits of propolis, several methods have been developed to remove allergenic compounds in propolis such as benzyl cinnamate and benzyl salicylate [50, 51]. However, the safety profile of purified propolis extracts has not been evaluated in individuals allergic to propolis yet.

### 3.2. Composition and Biological Activities of Royal Jelly

Royal jelly is an acidic, pungent smelling, yellow-whitish, creamy substance excreted from the pharyngeal gland of young *Apis mellifera* bee workers [13, 52, 53]. RJ is a complex material (Figure 2). In its fresh state, RJ consists largely of water (50%–60%) [54]. Proteins are the largest fraction in RJ (18%), and they represent around 50% of its dry weight [54]. Proteins in RJ are one of its most active components, especially a group of 9 nonwater-soluble proteins of molecular weights that range between 49 and 87 kDa, known as major RJ proteins (MRJPs1-9). MRJPs account for around 80% of the protein fraction of RJ, and MRJPs1-5 constitute 82-90% of MRJPs. MRJP1 is the most abundant among all MRJPs [37, 55, 56]. Due to their high content of amino acids (up to 578), MRJPs are reported to prevent senescence of human cells in vitro [57] as they promote cell proliferation, cell adhesion, cell growth, and immunity [58, 59]. The rest of the protein fraction of RJ comprises antioxidative peptides, free amino acids (including at least eight essential amino acids) [55], and small amounts of royalisin, jelleines, and aspimin—proteins, which express strong antimicrobial and bactericidal activities even against drug resistant bacteria [31, 32].

Carbohydrates constitute 15% of RJ content, and they consist mainly of glucose and fructose [54]. Sugars of RJ are thought to contribute to larval development into queens through stimulation of the activities of the nutrient sensing mammalian target of rapamycin pathway and insulin/insulin-like signaling, which stimulate food consumption and increase of body size [60]. Fats represent 3%–6% of RJ content [54], and they largely consist of a group of rare short hydroxyl fatty acids or dicarboxylic acids with 8–12 carbon atoms in the chain such as trans-10-hydroxy-2-decenoic acid (10-HDA, also known as queen bee acid or RJ acid) and sebacic acid. 10-HDA is one of the most common bioactive ingredients in RJ, and it is used as an indicator of its freshness [31, 61]. Furthermore, RJ is rich in phenols, vitamins (especially niacin and B complex), minerals, and trace elements. It also contains small amounts of nucleotides (e.g., adenosine, guanosine, and adenosine monophosphate), several bioactive substances (e.g., acetylcholine), volatile organic compounds (e.g., acetic acid, esters, and aldehydes), ketones, alcohol, and other compounds that have not been identified yet [37, 54, 55]. The percentages of compounds in RJ are relative, and they vary considerably according to several factors including bee species, geographical location, season, botanical origin, and sugar suplementation to nurse bees [37, 62].

Existing knowledge denotes that RJ is promising as an antiaging compound that is capable of enhancing health span through the promotion of reviving cellular processes such as cellular metabolism, protein translation, ribosomal biogenesis, and autophagy [37]. Owing to its antioxidant, anti-inflammatory, neuroprotective, cardioprotective, antiproliferative, antimicrobial, antilipidemic, antidiabetogenic, antiadipogenic, and antifatigue properties, RJ is used as an adjuvant modality for the treatment of various clinical conditions such as cancer, hypertension, hyperlipidemia, diabetes, and neurodegenerative diseases such as Alzheimer's disease and Parkinson's disease [37, 58, 63–71].

RJ is heat sensitive, and it should be kept frozen in order to keep its bioactive ingredients and pharmacological effects. This is because the storage of RJ at a temperature of 5°C or above promotes enzymatic degradation leading to loss of its content of soluble nitrogen and free amino acids [53]. Despite the fact that RJ has the potential to suppress histamine H1 receptor resulting in several antiallergic activities [30, 72], the occurrence of allergic reactions to RJ in people with a history of bronchial asthma and atopy has been reported in a number of case studies [73, 74]. Research demonstrates that some proteins in RJ are allergenic such as major royal jelly proteins 8 and 9 [37] and water-soluble proteins 1 and 2 (molecular weight: 55 and 47 kDa) [75]. Treating RJ with bacterial alkaline proteases removes allergenic proteins by breaking them into small peptides and amino acids without affecting RJ freshness or content of 10-HDA and vitamins [75, 76]. Administering proteases-treated RJ (pRJ) to individuals allergic to RJ did not cause allergic reactions in vivo and in vitro [76].

## 4. Animal Models of Parkinson's Disease

**Figure 3:**
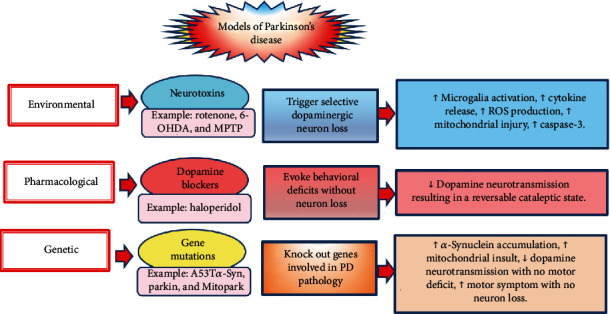
Various experimental models of Parkinson's disease. Multiple experimental models of PD have been developed. They are distinguished into 3 classes. Environmental models of PD are produced by numerous neurotoxins, which selectively induce dopaminergic neurodegeneration via mechanisms that involve induction of neurodegeneration through increased production of free radicals, inflammation, and mitochondrial dysfunction. Pharmacological models produce clinical manifestations of PD, e.g., catalepsy and dyskinesia without alterations in neuron structure or function—they block dopamine activity. Genetic models are miscellaneous, and they are produced through induction of gene mutations related to dopamine neurotransmission, mitochondrial function, and protein misfolding (*α*-synuclein). ↑ denotes increase; ↓ denotes decrease; PD: Parkinson's disease; MPTP: 1-methyl-4-phenyl-1,2,3,6-tetrahydropyridine; 6-OHDA: 6-hydroxydopamine; ROS: reactive oxygen species; caspase-3: cysteine-aspartic acid protease 3.

Experimental models of PD are distinguished into 3 main classes: genetic, pharmacological, and environmental (Figure 3). Various models express PD characteristics only partially, i.e., there is no typical model that perfectly exhibits the typical progressive, behavioral, neurologic, and pathological features of the disease, which may represent a challenge for PD research [6]. Many transgenic PD mice models have been recently generated by inducing mutations in genes involved in PD pathology such as A53T*α*-Syn, Thy-*α*-Syn, and aggregates of *α*-Syn preformed fibrils (PFF). These mutations accelerate intraneuronal accumulation of *α*-synuclein in a fashion similar to the naturally occurring ones [6, 77]. For instance, A53T*α*-Syn model develops mid- to late-onset neurodegeneration with aggregation of filamentous *α*-synuclein cytoplasmic inclusions all over the neuroaxis [77]. Some genetic models may demonstrate behavioral dysfunction without neurodegeneration (e.g., parkin, PINK1, and DJ-1) or demonstrate impaired dopaminergic transmission with no clear motor dysfunction (e.g., Leucine-rich repeat kinase 2 (LRRK2)). On the other hand, mice with mutations in vacuolar protein sorting-associated protein (VPS) 35 demonstrate aggregation of *α*-synuclein accumulation along with low striatal dopamine level and motor dysfunction. Mitopark models progressively develop motor deficits of PD due to mitochondrial insult in dopaminergic neurons resulting from knock out of the gene coding for mitochondrial transcription factor A [6].

Pharmacological treatments such as haloperidol block dopamine activity without inducing dopaminergic neurodegeneration in the SNC. Thus, they do not cause PD, but they induce transient behavioral alterations similar to those occurring in PD, known as PD akinetic deficit/akinesia or catalepsy [78]. Environmental models of PD are produced by treating animals with neurotoxins (e.g., rotenone), which selectively induce degeneration in dopaminergic neurons in the SNC via various mechanisms that embroil activation of signaling pathways involved in inflammation, oxidative stress, and apoptosis [79–81].

According to Table 1, PD models in studies included in this review were induced by treating laboratory animals and cell lines with 3 neurotoxins. Rotenone was used in one study to induce PD in rodents via inhibition of mitochondrial complex I of the mitochondrial respiratory chain, which was associated with high emission of mitochondrial ROS and induction of apoptosis via activation of caspase-3 [44]. 6-Hydroxydopamine (6-OHDA) has been used both in vivo and in vitro in 6 studies to induce PD [13, 42, 45, 79, 82, 83]. It selectively provokes apoptosis of dopaminergic neurons via mechanisms that involve increasing the production of ROS, accelerating lipid peroxidation (production of malondialdehyde (MDA)), and evoking an imbalance between the antiapoptotic B cell lymphoma 2 (bcl-2) and the proapoptotic bcl-2-associated X protein (bax) [79].

1-Methyl-4-phenyl-1,2,3,6-tetrahydropyridine (MPTP) and its metabolite 1-methyl-4-phenylpyridinium (MPP) were used in 3 studies to induce in vivo and in vitro models of PD [84–86]. Inside the body, MPTP changes into MPP, which activates key inflammatory signaling pathways such as nuclear factor kappa B (NF-*κ*B), interleukin-1, and inducible nitric oxide synthase (iNOS) resulting in SNC microglial activation, cytokine production, and secondary dopaminergic neuron damage [84, 85, 87]. In addition, MPTP moves into and accumulates inside nigrostriatal neurons through the activity of dopamine transporter while the membrane potential facilitates its transport into the mitochondria [85]. Accordingly, it alters mitochondrial enzymes resulting in increased mitochondrial production of ROS, collapse of mitochondrial membrane proteins, and mitochondrial membrane permeability. Mitochondrial membrane permeability promotes the leak of mitochondrial proteins (e.g., cytochrome c) into the cytoplasm, which is associated with activation of caspase-3, a downstream executive of apoptosis and cell death [84–86].

## 5. Evidence from Preclinical Studies

The effects of both propolis and its flavonoids and RJ and its lipids on PD were examined both in vivo and in vitro. The findings indicate that these bee products can induce both structural and symptomatic improvements and reduce the behavioral and histomorphometrical dysfunctions that are caused by PD in rodents. In this regard, a water extract of propolis (200 mg/kg/d/40 days) reduced dopaminergic neurodegeneration and striatal fiber degeneration induced by 6-OHDA along with maintenance of body weight and improvement of cardiac and autonomic functions [83]. CAPE (10 mg/kg) significantly decreased stepping ratio, improved coordination, shortened the latency to orient downwards on pole test, prolonged the permanence time, and increased the activity index and rears in rotenone-challenged mice [44]. This effect is a read out for reduced neurotoxicity demonstrated by CAPE in mice treated with MPTP and rotenone as depicted by increased percentage of viable dopaminergic neurons in the SNC by 73% and 92%, respectively [44, 84]. Chrysin treatment for 28 days protected mice against behavioral deficits triggered by interstriatal injection of 6-OHDA 36 days after lesion induction as reported by decreasing the number of rotations and latency for the first fall elicited by 6-OHDA. This report speaks for the molecular actions of chrysin, which contribute to neuronal survival such as increased production of neurotrophins and endogenous antioxidants reactivity and reduced levels of cytokines [42]. Moreover, treating 6-OHDA-PD rat models with RJ (100 or 200 mg/kg), 4 weeks after lesion induction, resulted in a significant decrease in the number of rotations in ipsilateral and contralateral to striatal lesion induced by apomorphine subcutaneous injection (0.2 mg/kg) in the 7th week after lesion induction compared with untreated PD rats [82]. The reported improvements of motor performance occurred for the reason that RJ restored the brain structure in 6-OHDA-treated rats by preventing neuron death in the SNC and in the caudate putamen unit (CPU) as well as by increasing the thickness of gray and white matter of the cerebral cortex and cerebellum compared with the untreated controls [82]. Pinocembrin (PB) was used only in vivo to evoke cellular and molecular effects, which are addressed in detail in Section 6 [79, 85, 86].  Table 2 presents more details on propolis and RJ treatments and key findings of the relevant studies.

## 6. Mechanisms of Action of Propolis and Royal Jelly in Parkinson's Disease

**Figure 4:**
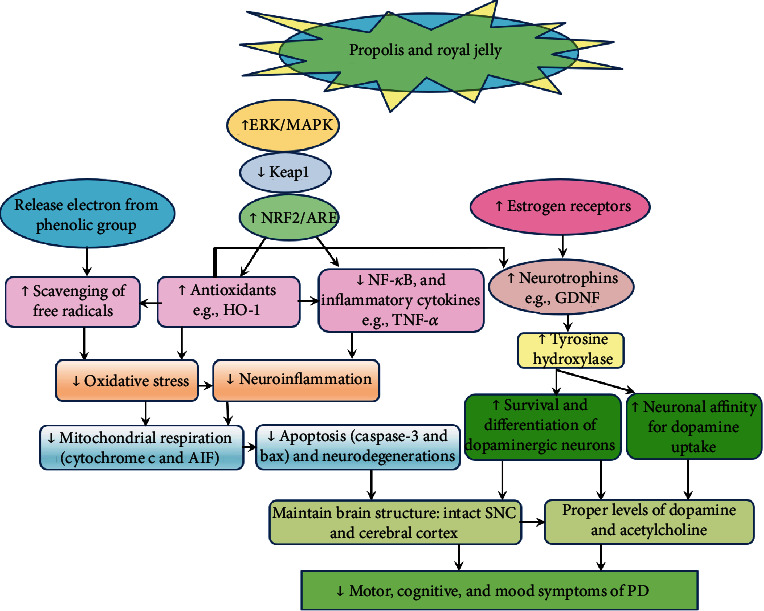
Probable mechanisms through which propolis and royal jelly (RJ) alleviate symptoms of Parkinson's disease. Propolis, RJ, and their compounds alleviate oxidative damage directly by scavenging free radicals through the release of an electron from their phenolic group and indirectly through activation of ERK/MAPK signaling, which deactivates Keap1, the molecule that degrades NRF2 resulting in NRF2 translocation into the nucleus to activate ARE, which stimulates the expression of antioxidant genes such as HO-1. On the other hand, NRF2 and HO-1 prevent the transcription of inflammatory pathways such as NF-*κ*B resulting in less production of inflammatory cytokines. Mitigation of oxidative stress and neuroinflammation is associated with less mitochondrial respiration and less production of apoptotic molecules such as caspase-3 and bax, eventually leading to less neurodegeneration. On the other side, chrysin increased the expression of various neurotrophic factors possibly through its contribution to HO-1 production; however, the detailed mechanism is not clearly understood. RJ also stimulates the expression of cerebral and hippocampal GDNF, possibly through activation of estrogen receptors. GDNF is associated with neuroprotective effects: enhancing the survival and morphological differentiation of midbrain dopaminergic neurons and fostering their affinity for dopamine. All these events prevent neuronal degeneration, maintain intact brain structure, keep proper levels of dopamine and acetylcholine, and eventually improve motor and cognitive symptoms of PD. ↑ denotes increase; ↓ denotes decrease; ERK: extracellular signal-regulated kinase; MAPK: mitogen-activated protein kinase; NRF2: nuclear factor erythroid 2; ARE: antioxidant response element; HO-1: heme oxygenease-1; NF-*κ*B: nuclear factor kappa B; TNF-*α*: tumor necrosis factor alpha; GDNF: glial cell line-derived neurotrophic factor; AIF: apoptosis inducing factor; caspase-3: cysteine-aspartic acid protease 3; bax: bcl-2-associated X protein; SNC: substantia nigra pars compacta; PD: Parkinson's disease.

Relatively few studies have explored the mechanism through which propolis and RJ may be beneficial for PD.  Figure 4 shows that propolis and RJ operate through a range of interrelated mechanisms, which are detailed in this section.

### 6.1. Bee Products Protect Neurons against Oxidative Stress

Antioxidants protect neurons against neurotoxins by inhibiting the generation of free radicals. Several lines of evidence denote that flavonoids in propolis and derivatives of RJ lipids demonstrate neuroprotective effects in dopaminergic neurons, to a great extent, through modulation of oxidative stress. CAPE blocked the production of O_2−_ and peroxynitrite in the brain of MPTP-intoxicated mice and inhibited the activity of the prooxidant iNOS in rotenone-induced mouse model of PD [44, 84]. In vitro investigations show that CAPE also protected cerebellar granule neurons (CGNs) and rostral mesencephalic neurons (RMNs) against free radicals induced by 6-OHDA [45, 88]. PB conferred protection against cytotoxicity induced by MPP+ and 6-OHDA in vitro through upregulation of the expression of heme oxygenase-1 (HO-1) [85, 86], superoxide dismutase (SOD), and *γ*-glutamylcysteine synthetase (*γ*-GCS) [79]. An in vitro study involving application of 6-OHDA to human neuroblastoma SH-SY5Y cells to induce cell death as a model of PD showed that treatment with a derivative of RJ lipids known as 4-hydroperoxy-2-decenoic acid ethyl ester (HPO-DAEE) markedly stimulated the expression of antioxidant genes such as HO-1, *γ*-glutamylcysteine ligase (*γ*-GCL), and NAD(P)H quinone dehydrogenase 1 (NQO1) [13].

Antioxidants such as HO-1, which act as stress-related/phase II detoxification enzymes, are thought to have a role in PD pathology. This suggestion is derived from the fact that HO-1 is upregulated in the cytoplasm of dopaminergic neurons in the SNC of PD brains while the saliva of PD patients expresses a moderate increase in HO-1 protein levels compared with normal cross-matched control individuals. In addition, Lewy bodies exhibit a strong immunoreactivity to HO-1 [89, 90]. On the other hand, injecting adenovirus containing human HO-1 gene (Ad-HO-1) locally into the SNC of rats intoxicated with MPP+ significantly accelerated the survival rate of dopaminergic neurons—an effect that was associated with upregulation of brain-derived neurotrophic factor (BDNF) and glial cell line-derived neurotrophic factor (GDNF), downregulation of tumor necrosis factor-*α* (TNF-*α*) and interleukin-1*β* (IL-1*β*), and restoration of dopamine levels in the SNC. Ad-HO-1 counteracted apomorphine-induced rotation following MPP+ treatment. Hence, increased levels of HO-1 in PD brains represent a defense attempt against neuronal insult [91]. In this regard, Wang et al. [86] reported that treating mice with MPP+ induced HO-1 in a dose-dependent fashion. Cotreatment with PB further accelerated the expression of HO-1, which was associated with significant reductions in MPP+-induced neurotoxicity, ROS production, cleavage of caspase-3, and the rate of apoptosis and cell death in a dose-dependent manner [79, 85, 86]. Inhibition of HO-1 by zinc protoporphyrin-IX attenuated the neuroprotective effects of PB [86].

Western blot analysis shows that the antioxidant effects of PB and HPO-DAEE in SH-SY5Y cells result from activation of 2 main signaling pathways: nuclear factor erythroid 2- (NRF2-) antioxidant response element (ARE), which is a main regulator of antioxidative responses, and eukaryotic initiation factor 2*α* (eIF2*α*), an upstream effector of the activating transcription factor-4 (ATF4), which cooperates with NRF2 to modulate the expression of antioxidant genes [13, 86]. Interestingly, the mechanism through which HPO-DAEE exerts its antioxidant effect involves slight emission of ROS upon HPO-DAEE treatment, which represents a sublethal stress that causes preconditioning of SH-SY5Y cells against subsequent severer oxidative stress induced by 6-OHDA. The authors noticed that ROS deactivated Keap1, the cytoplasmic protein that degrades NRF2, which allowed NRF2 phosphorylation and translocation into the nucleus. In the nucleus, NRF2 binds to the small Maf proteins to form heterodimers, which further phosphorylate ARE in the 5′-regulatory region of antioxidant enzymes and phase II detoxifying enzymes—which result in the expression of antioxidant genes. In the same manner, HPO-DAEE activated the phosphorylation of eIF2*α*, which resulted in aggregation of ATF4 in the nucleus, and subsequent augmentation of NRF2-induced activation of the HO-1 expression, which eventually protected cells against 6-OHDA toxicity [13]. The mechanism involved in NRF2 activation by PB is a bit different.

In vitro investigations revealed that PB induced the phosphorylation of extracellular signal-regulated kinase (ERK) 1 and 2/mitogen-activated protein kinase (MAPK) in SH-SY5Y cells treated with neurotoxins [86] resulting in deactivation of Keap1 and phosphorylation of NRF2 [86] and ARE [79], which caused a significant increase in the production of antioxidant enzymes [79, 85, 86]. Suppression of ERK/MAPK signaling by PD98059, an ERK inhibitor, or suppression of HO-1 by zinc protoporphyrin abolished the neuroprotective effects of PB [86]. Similarly, knockdown of the NRF2 expression by transfecting SH-SY5Y with scrambled NRF2 or NRF2-specific small interfering RNA (siRNA) inhibited the antioxidant effect of PB: these maneuvers inhibited the expression of NRF2 and its target genes (HO-1 and *γ*-GCS), which accelerated 6-OHDA-induced cell death [79]. Altogether, these findings confirm that the PB activates ERK/MAPK/NRF2/ARE cascade resulting in the production of endogenous antioxidants, which in turn suppress neurotoxicity and apoptosis [79, 85, 86].

### 6.2. Bee Products Protect Neurons against Neuroinflammation

Research shows that both central and local inflammation, which involves CD4 T cell infiltration and activation of CD11b+microglia/macrophages, play a key role in neuron loss in PD. Chronic activation of these cells is associated with morphological and functional alterations that promote excessive production of ROS [44, 87]. According to Table 2, treating a rotenone-induced mouse model of PD with CAPE (2.5-10 mg/kg/every other d/17 days) inhibited microglial activation (CD11b+); downregulated the activity of NF-*κ*B, iNOS, and cyclooxygenase-2 (COX-2); and reduced the production of TNF-*α* and IL-1*β*. Amelioration of rotenone-induced inflammatory response was associated with increased dopaminergic neuronal survival and decreased motor deficits [44]. Likewise, oral treatment of rats intoxicated by 6-OHDA with chrysin (10 mg/kg/twice a day) inhibited NF-*κ*B signaling, which was accompanied by downregulation of inflammatory markers (e.g., TNF-*α*, INF-*γ*, IL-1*β*, IL-6, IL-10) and related destructive molecules such as total reactive antioxidant potential (TRAP) and calcium-binding protein B (S100B) [42].

Research shows that bee products such as RJ display immunomodulatory and anti-inflammatory functions under conditions of neuroinflammation via activation of NRF2 [13]. In addition to being a master pathway that stimulates the release of antioxidants, NRF2 plays a central role in the suppression of inflammatory responses directly through downregulation of the transcription of proinflammatory cytokines such as IL-6 and IL-1*β* [92]. Moreover, redox control (expression of antioxidant genes such as HO-1) is another mechanism through which RJ might silence neuroinflammation [13]. HO-1 is a main cytoprotective agent not only against oxidative stress but also against inflammation. It exerts its anti-inflammatory effect by catalyzing the enzymatic degradation of nonprotein bound free heme, which demonstrates cytotoxic and proinflammatory properties. Heme degradation induced by HO-1 results in three main catabolites: [1] carbon monoxide, which expresses numerous anti-inflammatory and antiapoptotic effects, [2] ferritin, which chelates free iron, and [3] biliverdin, which is reduced by biliverdin reductase into bilirubin, a potent antioxidant [93]. Activation of HO-1 gene in H_2_O_2_-challenged human neuroblastoma cells SH-SY5Y treated by Carvacrol (an essential oil found in Labiatae) is associated with downregulation of NF-*κ*B, low lipid peroxidation, and less carbonylation and nitration of proteins of the mitochondrial membrane [94].

### 6.3. Inhibiting Apoptosis of Dopaminergic Neurons

Cell viability assays [13, 45, 79, 84–86, 88] and tyrosine hydroxylase (TH) immunohistochemistry analysis were used in many studies to examine dopaminergic neurons loss in the SNC [42, 44, 81, 84]. These investigations showed that relatively high doses of CAPE (5 or 10 mg/kg) decreased neurotoxicity induced by MPTP and rotenone as demonstrated by the increased percentage of TH-immunostained neurons in the SNC by 73% and 92%, respectively [44, 84]. Four-week treatment of 6-OHDA-induced rat models of PD with chrysin resulted in a significant increase in the number of TH+ neurons in the SNC compared with untreated animals [42]. The neuroprotective effects of CAPE [45, 84, 88], PB [79, 85, 86], and HPO-DAEE [13] were vividly expressed in CGNs, RMNs, and SH-SY5Y cells that were challenged with neurotoxins.

Loss of dopaminergic neurons in PD is attributed, at least in part, to activation of various signaling pathways (e.g., Jun N-terminal kinase), which induce apoptosis as a programmed form of morphological cell death in PD. Neurotoxins such as MPTP induce PD via activation of Jun N-terminal kinase [95, 96]. Therefore, the neuroprotective effects of phenolic compounds in propolis as well as RJ and its derivatives, which were observed in different models of PD (Table 2), might be due to modulation of cell survival and apoptotic pathways.

Active flavonoids in propolis inhibited dopaminergic neuronal apoptosis by affecting the signaling of mitochondrial apoptotic pathway. PB remarkably inhibited the cleavage of caspase-3 and decreased the rate of apoptosis by blocking MPP+-induced mitochondrial alterations, e.g., mitochondrial release of cytochrome c and reduction of membrane potential, thus preventing the transfer of MPP+ into mitochondria; thus, inhibiting ROS production and mitochondrial membrane permeability, which evoke apoptotic pathways [85]. In the same way, CAPE increased the survival of 6-OHDA-treated CGNs via a mechanism that involved stabilizing mitochondrial functioning. It mitigated apoptosis by deactivating caspase-3 and inhibiting mitochondrial release of cytochrome c under treatment with Ca_2+_ [45].The antiapoptotic effects of CAPE are possibly related to amelioration of oxidative stress. In vivo, CAPE inhibited the activity of iNOS and caspase-1 in the brain of mice treated with MPTP [84]. To explore the detailed mechanism, the authors isolated brain mitochondria and treated them with MPP, which is known to stimulate ROS production. MPP increased the production of ROS, cytochrome c, and apoptosis inducing factor (AIF), which mediate MPP neurotoxicity. CAPE inhibited mitochondrial release of cytochrome c and AIF [84]. Whereas neurotoxins such as MPTP trigger cell death signaling via increasing bax and inhibiting bcl-2, the effect of bee products and their derivatives (e.g., PB) on dopaminergic cell viability is mediated by regulating the expression of bax and bcl-2 in an antiapoptotic mechanism [85].

### 6.4. Maintaining Brain Levels of Dopamine

Dopamine is the main neuroactive substance involved in PD [31]. Current PD treatments are based on dopamine replacement [44]. Active compounds in propolis increased dopamine levels in the SNC of experimental models of PD. In this regard, treatment of rats intoxicated by 6-OHDA with chrysin (10 mg/kg/twice a day) increased the levels of dopamine, 3,4-dihydroxyphenylacetic acid (DOPAC), and homovanillic acid (HVA) [42]. DOPAC and HVA portray dopamine turnover in dopaminergic nerve terminals [35]. In the same way, high-performance liquid chromatography showed that CAPE (10 mg/kg) increased striatal levels of dopamine in MPTT-PD mice [44, 84]. CAPE did not alter mono amino oxidase activity and brain levels of MPP denoting that its neuroprotective activity does not involve decreasing the metabolism of MPTP to MPP+ [84]. Therefore, enhanced dopamine production following treatment with chrysin [42] and CAPE [44, 84] speaks for the neuroprotective effects of these compounds—as noted by viability assays and neurohistochemistry analyses, which revealed improved neuronal viability and increased numbers of TH+ neurons in animals challenged with neurotoxins following treatment with RJ, HPO-DAEE, and flavonoids of propolis (Table 2).

The contribution of RJ, CAPE, and chrysin to maintenance of the dopaminergic system in PD is probably linked to their antioxidant activity and the associated anti-inflammatory and antiapoptotic effects [31, 42, 44, 45, 79, 85, 86, 88]. Dopamine is a monoamine derived from tyrosine. Environmental factors, including the consumption of food rich in tyrosine, can boost the production of dopamine [82]. RJ contains tyrosine in addition to numerous free amino acids, e.g., valine, glutamic acid, serine, glycine, cysteine, threonine, alanine, tyrosine, phenylalanine, hydroxyproline, leucine-isoleucine, and glutamine. Some of these amino acids can be converted into tyrosine [31, 97]. Thus, RJ may promote the biosynthesis of dopamine [31]. Queen bees exhibit higher brain levels of dopamine and its metabolites (e.g., N-acetyl-dopamine and norepinephrine) than workers [98]. Meanwhile, treating workers with RJ stimulated the gene expression of several types of receptors of dopamine and tyramine [98]. Evidence indicates that the consumption of both RJ and tyrosine significantly improves brain levels of dopamine, tyramine, and their metabolites in emerging bee workers (4-8 days old) and in 8 days old males compared with control bees. The author indicated a relationship between age and the dopaminergic effect of RJ [97, 98]. In line, oral supplementation of RJ to mice treated with tartrazine—an azo dye derived from coal tar that has a DNA-damaging effect—was associated with reduction of apoptotic cell markers and restoration of different brain neurotransmitters, including dopamine [99]. RJ contains exceptionally high concentrations of acetylcholine (4–8 mM), which resists degradation due to the acidic pH (4.0) of RJ. Hence, the well-conserved acetylcholine of RJ [100] might positively contribute to neuromodulation in the dopamine-acetylcholine circuit of PD. Evidence denotes that agonism of *α*7 nicotinic acetylcholine receptors in the CNS provides maximal beneficial responses with minimal adverse effects since it prevents neuronal cell loss in the SNC and reduces levodopa-induced dyskinesias [101]. Further in-depth investigations of the molecular events involved in dopamine increase following treatment with bee products are needed.

### 6.5. Enhancement of the Production of Neurotrophins in the Brain

Neuronal adversities such as chronic oxidative stress, neuroinflammation, and excitotoxicity are key contributors to progressive neurotoxicity and neurodegeneration. Neurotrophins are compounds that are essential for the survival of specific neurochemical phenotype classes of neurons [42]. As illustrated in Table 2, only one study examined the effect of bee products on the production of neurotrophins. Chrysin increased the expression of BDNF, nerve growth factor (NGF), and GDNF in 6-OHDA-PD mice [42]. Given that induction of HO-1 in MPP+ models via Ad-HO-1 is associated with increased production of BDNF and GDNF in the SNC [91], it is possible that the neurotrophic effects of chrysin are triggered by its antioxidant action. However, the definite underlying molecular events are not clear.

GDNF, one of the main neurotrophic factors of the brain, is a distantly related member of the transforming growth factor-beta superfamily [102]. Research signifies a role of GDNF in the treatment of PD: it maintains the survival and morphological differentiation of cultured midbrain dopaminergic neurons and enhances their dopamine uptake [103]. Meanwhile, injecting external GDNF into the SNC or striatum of aged mice experiencing a partial 6-OHDA-induced lesion increases levels of tyrosine hydroxylase mRNA in lesioned dopaminergic neurons, which is associated with less degeneration of nigrostriatal dopaminergic neurons and increased affinity of binding of the dopaminergic transporter ligand [(125)I]IPCIT in the lesioned striatum [104]. In another study, oral consumption of RJ by adult mice stimulated the expression of GDNF [102], which implies that the neuroprotective effect of RJ in dopaminergic neurons could be attributed to enhancing GDNF levels. RJ and its fatty acids demonstrate an estrogenic activity as they bind estrogen receptors *β* and *α* to stimulate the release of BDNF, GDNF, and NGF. These neurotrophins modulate cell proliferation and regulate the expression of various genes that counteract inflammation and oxidative stress in the brain neurons [61, 66, 105–107].

### 6.6. Restoration of Normal Brain Structure

PD involves morphological alterations in different parts of the brain, including reduced volumes of the caudate nucleus, thalamus, and white matter, as well as atrophy of the basal ganglia, contraction in the left cerebellum, decreased gray matter in the right quadrangular lobe, reduced fractional anisotropy, neuromelanin pigmentation, neuronal loss within the SNC, and increased mean and radial diffusivity within the SNC and globus pallidus [108]. Experimental models indicate that RJ induces structural and symptomatic improvements in PD mice by protecting against the histomorphometrical dysfunctions caused by the disease. In this context, one study examined the effect of oral RJ treatment (100 or 200 mg/kg/day/3 weeks) on the brain structure of male mice receiving unilateral injection of 6-OHDA in the CPU. Histomorphometry revealed that compared with untreated PD mice, the numbers of Nissl-stained neurons in the SNC and CPU were significantly higher in the RJ (100 or 200 mg) treated groups. Both doses of RJ significantly increased the thickness of gray and white matter of the cerebral cortex and cerebellum [82]. Similarly, light microscopical examination of the cerebral cortex structure in mice treated with tartrazine revealed less damages in animals receiving RJ compared with controls as indicated by fewer cells with pyknotic nuclei as well as less ssDNA positive cells [99]. The effect of RJ on the integrity of brain structure is attributed to its antioxidant, anti-inflammatory, and antiapoptotic effects, which all lower the loss of dopaminergic and cholinergic neurons [99, 109]. The effect of propolis on brain structure was evaluated in 6-OHDA-challenged rats. Compared with the sham and placebo (water) groups, propolis significantly reduced striatal fiber degeneration [83]. However, the effect of active compounds in propolis on anatomical structures in the brain needs to be explored in future studies.

## 7. Discussion

Few preclinical studies have evaluated the effect of propolis flavonoids, RJ, and RJ lipids on PD. Whole RJ [82], CAPE [44], and chrysin [42] improved motor behavioral alterations in PD animals. The protective effects of these compounds are likely attributed to their ability to reduce the production of free radicals [13, 42, 44, 45, 79, 84, 86], proinflammatory cytokines [42, 44], and mitochondrial proteins [45, 84] involved in cell death as well as their downstream effectors such as caspase-3 [85] and bax [79]. Immunochemistry and cell viability analyses revealed higher survival of dopaminergic neurons treated with these compounds both in vivo [42, 44, 84] and in vitro [13, 45, 79, 84, 86]. These results indicate that bee products such as propolis and RJ can be a potentially safe adjunctive treatment for PD.

Investment in animal studies provides valuable knowledge, which sometimes cannot be derived from human trials due to ethical, technical, and logistic complexities. To our knowledge, the effect of propolis and RJ on PD in humans has not been examined till now. Yet, considering the reported anti-Parkinson effects of these products in laboratory animals, similar effects might be expected to occur in humans. Therefore, the anti-Parkinson effects of propolis and RJ deserve to be evaluated in soundly designed RCTs.

From a theoretical point of view, propolis and RJ may be beneficial not only for the main motor symptoms of PD but also for other nonmotor symptoms such as cardiac, autonomic, GI, depressive, and cognitive symptoms [19, 83, 110]. In connection with this standpoint, propolis (200 mg/kg/40 days) increased the survival of dopaminergic neurons and maintained striatal structure in a PD rat model induced by bilateral injection of 6-OHDA in the striatum. Along with such neurological improvements, propolis also restored body weight and reverted the cardiological and autonomic alterations caused by 6-OHDA treatment as detected by increased heart rate and heart rate variability compared with the placebo treatment [83]. Existing knowledge emphasizes an association between PD and cardiac disorders as they share common pathologies such as inflammation, cellular stress, and impaired metabolism of lipids and glucose. Meanwhile, coffee consumption and physical activity seem to be protective against both conditions [111]. Common autonomic symptoms in PD patients (e.g., orthostatic hypotension) interfere with activities of daily living, limit physical activity, and heighten the risk of falls, hospitalization, and mortality [112]. Evidence denotes that autonomic failure in PD patients is significantly linked to striatal serotonergic and dopaminergic degeneration expressed by lower [123I]FP-CIT binding ratios in the right caudate nucleus. These symptoms are mostly driven by GI and cardiovascular dysfunctions [113].

RJ may relieve GI discomfort commonly experienced by most PD patients. Evidence indicates that RJ alleviates GI symptoms such as anorexia in cancer patients [114] and constipation [115]—its acetylcholine content exerts a direct effect on the muscarinic receptors of the intestinal smooth muscle resulting in intestinal contractions. However, this effect does not stem from a single oral dose [115]. From another perspective, evidence indicates that early development of dopaminergic lesions in a 6-OHDA rat model of PD is associated with reduction of BDNF and occurrence of depressive-like behaviors, which could be relieved by antidepressant treatment [116]. More, PD is known as manifest Lewy body dementia because severe cognitive alterations develop, especially during late stages of the disease [19]. Research shows that propolis and RJ possess a capacity to improve cognitive performance [71] and alleviate depressive symptoms through mitigation of oxidative stress [117] and activation of signaling pathways that promote the production of various neurotrophic factors such as BDNF [118], GDNF [102], and NGF [119, 120]. Altogether, according to these scenarios, propolis and RJ may represent a multidimensional treatment, which may promote the quality of life among PD patients.

It is now well-known that altered gut microbiome is a major contributor to the initiation and development of PD pathology [10, 11, 121]. Evolving knowledge implies that dietary interventions such as fatty acids (phospholipid membrane precursors), amino acids, and microbiota-directed therapy (e.g., probiotics, prebiotics, and postbiotics) may correct gut alterations, treat GI symptom, and promote CNS functioning in PD [10, 18, 121, 122]. Propolis and RJ are rich in amino acids, fatty acids, and phospholipids [28, 37, 41, 55]. In addition, they are abundant in beneficial bacteria such as lactic acid bacteria, which is commonly used as probiotics to improve health and enhance growth and reproductive performance—43 species of these bacteria have been identified in bees and bee products such as RJ with 20 of them having inhibitory effects against 28 species of human and animal pathogens, some of which are antibiotic-resistant [123]. Propolis possesses strong antimicrobial properties, and it is used by bee workers as a disinfecting agent to keep integrity of the beehive [40]. Meanwhile, RJ contributes to the diversity and vitality of gut microbiome in queen bees. For example, *Lactobacillus apis* and *Bifidobacterium* are abundant in the gut of queen bees, and they produce metabolites that prevent the expression of oxidative stress genes and contribute to the excellent physical and reproductive traits of queen bees. On the other hand, these bacteria are deficient in bee workers, which feast mainly on honey and pollens [124]. The literature denotes that both propolis and RJ contribute to the maintenance of GI function. In this respect, supplementing rats on high fat diet with 0.2% dietary green propolis significantly altered the structure of gut microbiome. This effect was associated with less intestinal permeability, lower levels of lipopolysaccharide in the systemic circulation, and downregulation of activity of toll-like receptor 4 and cytokine expression in skeletal muscle [125]. Propolis was also reported to protect rats against gastric mucosal lesions induced by stress [126]. RJ has been shown to enhance the growth of beneficial gut bacteria such as *Bacteroides fragillis* and *Bacteroides thetaiotaomicron*, which colonize in the distal end of the gut to ferment and degrade indigestible proteins and carbohydrates; they also play a role in the activation of the regulatory T cells. In addition, RJ also demonstrates protective effects on the intestinal wall by contributing to the viability of the human epithelial colorectal cell line Caco-2 [127]. Thus, we suggest that propolis and RJ may positively affect the gut-brain axis in PD patients by modulating microbiota composition. Future studies exploring the effect of bee products on microbiota in PD and its association with the molecular and cellular adversities associated with PD will provide insightful information. It might be helpful to compare the effect of combining propolis and RJ with other conventional treatments such as dietary modifications and exercise since these interventions express their effects, in part, through the modulation of gut microflora [128].

Identification of target subjects for RCTs using bee products to prevent pathologies underlying PD is of crucial importance. Since GI symptoms, especially constipation, occur in 80% of PD patients a long time before appearance of motor symptoms, early treatment of people with chronic constipation may represent a preventive modality of PD that worth investigation in future RCTs [10]. Various types of genetic variation may affect response to treatment with api-materials, which may be challenging if we are to identify candidates for pathology prevention in humans. For instance, Asian PD patients, not Caucasians, exhibit mutations in coiled-coil-helix-coiled-coil-helix domain containing 2, a mitochondrial protein that decreases oxygen consumption and ATP production, increases ROS production and mitochondrial cristae dilation resulting in heightened apoptotic degeneration in skeletal muscle. Immunosignals of this protein are most profuse in dopaminergic neurons of the SNC, cortical and hippocampal pyramidal neurons, and motor neurons in the anterior horn of the spinal cord [129]. In addition to race, possible other characteristics that might affect muscular functions should also be addressed such as gender, general health status, activity level, psychosocial stresses, nutrition, and levels of testosterone.

Both propolis and RJ comprise a large number of components. As shown in Table 2, CAPE, chrysin, and PB, which are key flavonoids in propolis, could modulate oxidative, inflammatory, and apoptotic events that underly the pathogenesis of PD. On the other hand, the literature is short of information on the most beneficial specific ingredients for PD in RJ [31, 130]. Yet, according to the current knowledge, RJ lipid component might be the most probable candidate—HPO-DAEE ameliorated oxidative stress in neurons treated with 6-OHDA [13]. On the other hand, MRJPs, peptides, and amino acids contribute, in a great part, to the multiple biological properties of RJ [58, 59]. Still, their role in PD has not been adequately explored. Similarly, the effect of RJ contents of tyrosine (a dopamine precursor) and acetylcholine (which function as a neurotransmitter) has not been examined in PD models. In addition, adenosine monophosphate N1 oxide (AMP N1-oxide) is another ingredient that merits further investigation given that it stimulates neuronal differentiation [131].

It is worth noting that natural products such as propolis and RJ can be affected by several factors such as season, geographical location, botanical source, bee species, environmental conditions (e.g., storage temperature), harvesting time (e.g., RJ harvested within 24 hours from larvae is higher in its phenols content than that harvested 72 hours or later), and methods of handling (e.g., pRJ contains more bioactive ingredients than crude RJ such as short peptides and amino acids) [31, 37, 130]. All such factors can affect the potency and quality of bioactive ingredients in these products. Hence, researchers should consider the relevant evidence concerning the quality of the used api-materials and their ingredients in intended RCTs. In the reviewed experimental studies, propolis extracts and RJ were administered orally; however, it is not clear if this route has the best effect. This is because, MRJPs, except for MRJP2, get rapidly degraded in the stomach and small intestine [132]. Therefore, attention should be paid to the route of administration, e.g., the absorption of capsulated RJ might be better than direct oral consumption.

## 8. Conclusion

All animal studies discussed above indicate that whole propolis, CAPE, PB, chrysin, whole RJ, and its lipid derivative (HPO-DAEE) might counteract oxidative stress, neuroinflammation, and mitochondrial dysfunction resulting in mitigation of neuronal damage and improvement of motor symptoms of PD. However, more studies are needed to examine the specific cellular and molecular mechanisms of whole propolis and RJ, as well as main compounds in RJ (MRJPs, peptides, phenols, bioactive substances) in PD. To identify the most effective compounds in propolis and RJ, it might be important to compare the effect of various active ingredients in these products either alone or in combination with other PD treatments. Future RCTs that investigate the effect of bee products on PD should consider individual variations (e.g., race, gender, general health, activity level, and diet) and accompany subjective outcome measures with sound predictive biomarkers.

## Figures and Tables

**Table 1:** Neurotoxins used to induce Parkinson's disease in animal models and cell lines in included studies.

Neurotoxin	Treatment	Mechanism of neurotoxicity	Reference
Rotenone	In vivo.	↓ Mitochondrial complex I. ↑ ROS and caspase-3.	[44]
MPTP and MPP	In vivo and in vitro.	↑ ROS, production of cytokines, and caspase-3.	[84–86]
6-OHDA	In vivo and in vitro.	↑ ROS, MDA, caspase-3, bax. ↓ Bcl-2 and SOD.	[13, 42, 45, 79, 82, 83]

MPTP: 1-methyl-4-phenyl-1,2,3,6-tetrahydropyridine; MPP: 1-methyl-4-phenylpyridinium; 6-OHDA: 6-hydroxydopamine; bcl-2: B cell lymphoma 2; bax: bcl-2-associated X protein; ROS: reactive oxygen species; MDA: malondialdehyde; SOD: super oxide dismutase; caspase-3: cysteine-aspartic acid protease 3.

**Table 2:** Characteristics of included preclinical experiments involving administration of propolis, royal jelly, and their constituents in animal and cell culture models of Parkinson's disease (number of included studies = 11).

Animal/cell line model	Treatment	Summary of effects and mechanism	Reference
6-OHDA-induced rat model of PD	Propolis gavage (200 mg/kg/d/40 days)	↑ TH+ neurons ↓ Striatal fiber degeneration Propolis restored body weight and reverted 6-OHDA-induced reductions in HR and HRV	[83]
Rotenone-induced mouse model of PD	CAPE (2.5-10 mg/kg/p.o./every other d/17 days)	↓ Motor deficits, microglia activation (CD11b+), TNF-*α* and IL-1*β*, COX-2, iNOS, and NF-*κ*B ↑ Striatal dopamine and TH+ neurons	[44]
MPTP-induced mouse model of PD MPTP-treated CGNs and RMNs as cellular models of PD	CAPE (2-10 mg/kg/p.o./d/7 days)	↑ Neuronal viability, striatal dopamine, TH+ neurons ↓ iNOS, caspase-1, cytochrome c, AIF, MPP-induced formation of free radicals and peroxynitrite, and neurotoxicity	[84]
6-OHDA-treated CGNs and hepatic cells as a cellular model of PD and oxidative stress	CAPE (10 *μ*M/4 h) followed by a treatment with 6-OHDA (70 *μ*M for 6 h)	↑ Neuronal viability ↓ Cytochrome c, caspase-3, and H_2_O_2_-induced neurotoxicity	[45]
6-OHDA-treated CGNs and RMNs as a cellular model of PD	CAPE (10 *μ*M) for 2 h	↑ Neuronal viability ↓ Formation of O_2_− and peroxynitrite and H_2_O_2_-induced neurotoxicity	[88]
MPP+-treated SH-SY5Y cells as a cellular model of PD	PB (1, 10, 20 *μ*M) for 24 h	↑ Neuronal viability, mitochondrial membrane potential, and bcl-2/bax ratio ↓ ROS, caspase-3, apoptotic rate, and cytochrome c	[85]
MPP+-treated SH-SY5Y cells as a cellular model of PD	PB (20 *μ*M) for 24 h	↓ Neurotoxicity ↑ ERK/MAPK and HO-1	[86]
6-OHDA-treated SH-SY5Y cells as a cellular model of PD	PB (25 *μ*M) for 4 h	↓ Neurotoxicity, ROS, MDA, and apoptotic rate ↑ Nrf2/ARE, SOD, HO-1, *γ*-GCS, bcl-2/bax ratio, and mitochondrial membrane potential	[79]
6-OHDA-induced rat model of PD	Chrysin orally (10 mg/kg/twice a d/28 days)	↓ TNF-*α*, INF-*γ*, IL-1*β*, IL-6, IL-10, NF-*κ*B, TRAP, and S100B ↑ Dopamine, DOPAC, HVA, BDNF, NGF, GDNF, TAR, and TH+ neurons	[42]
6-OHDA-induced mouse model of PD	Dietary RJ 100 or 200 mg (3 weeks)	↑ Neuronal survival (the number of Nissl-stained neurons in the SNC and CPU) and the thickness of gray and white matter of the cerebral cortex and cerebellum ↓ Motor deficits and contralateral to striatal lesion induced by apomorphine injection	[82]
6-OHDA-treated SH-SY5Y cells as a cellular model of PD	RJ fatty acids: 10-HDA, 10H2DA, SA, their derivatives DAEE and HPO-DAEE (4 h)	Only HPO-DAEE ↑ cell viability, NRF2-ARE signaling, HO-1, *γ*-GCL, and NQO1 ↓ ROS emission	[13]

↑ denotes increase; ↓ denotes decrease; RJ: royal jelly; d: day; h: hour; PD: Parkinson's disease; 6-OHDA: 6-hydroxydopamine; HR: heart rate; HRV: heart rate variability; SNC: substantia nigra pars compacta; CPU: caudate putamen unit; 10-HDA: 10-hydroxy-decanoic acid; 10H2DA: 10-hydroxy-2-decenoic acid; SA: sebacic acid; DAEE: 2-decenoic acid ethyl ester; HPO-DAEE: 4-hydroperoxy-2-decenoic acid ethyl ester; HO-1: heme oxygenase 1; *γ*-GCL: *γ*-glutamylcysteine ligase; NQO1: NAD(P)H quinone dehydrogenase 1; ROS: reactive oxygen species; NRF2-ARE: nuclear factor erythroid 2-antioxidant response element; eIF2*α*-ATF4: eukaryotic initiation factor 2, an upstream effector of the activating transcription factor-4; CAPE: caffeic acid phenethyl ester, genes encoding CD11b (a microglia surface antigen); COX-2: cyclooxygenase-2; iNOS: inducible nitric oxide synthase; NF-*κ*B: nuclear factor-*κ*B; TH: tyrosine hydroxylase; MPTP: 1-methyl-4-phenyl-1,2,3,6-tetrahydropyridine; CGNs: cerebellar granule neurons; RMNs: rostral mesencephalic neurons; p.o.: oral gavage; AIF: apoptosis inducing factor; MPP: 1-methyl-4-phenylpyridinium; caspase-3: cysteine-aspartic acid protease 3; H2O2: hydrogen peroxide; PB: pinocembrin; bcl-2: B cell lymphoma 2; bax: bcl-2-associated X protein; ERK: extracellular signal-regulated kinase; MAPK: mitogen-activated protein kinase; *γ*-GCS: *γ*-glutamylcysteine synthetase; MDA: malondialdehyde; SOD: super oxide dismutase; DOPAC: 3,4-dihydroxyphenylacetic acid; HVA: homovanillic acid; BDNF: brain-derived neurotrophic factor; NGF: nerve growth factor; GDNF: glial cell line-derived neurotrophic factor; TNF-*α*: tumor necrosis factor-*α*; INF-*γ*: interferon-gamma; IL-1*β*: interleukin-1*β*; IL-2: interleukin-2; IL-6: interleukin-6; IL-10: interleukin-10; NF-*κ*B: nuclear factor-kappa B; S100B: calcium-binding protein B; TRAP: total reactive antioxidant potential; TAR: total antioxidant reactivity.

## Abbreviations

6-OHDA: 6-Hydroxydopamine
10H2DA: 10-Hydroxy-2-decenoic acid
10-HDA: Trans-10-hydroxy-2-decenoic acid
*γ*-GCL: *γ*-Glutamylcysteine ligase
*γ*-GCS: *γ*-Glutamylcysteine synthetase
AIF: Apoptosis inducing factor
ARE: Antioxidant response element
ATF4: Activating transcription factor-4
Bax: Bcl-2-associated X protein
Bcl-2: B cell lymphoma 2
BDNF: Brain-derived neurotrophic factor
CAPE: Caffeic acid phenethyl ester
Caspase-3: Cysteine-aspartic acid protease 3
CGNs: Cerebellar granule neurons
CNS: Central nervous system
COX-2: Cyclooxygenase-2
CPU: Caudate putamen unit
DOPAC: 3,4-dihydroxyphenylacetic acid
DAEE: 2-Decenoic acid ethyl ester
eIF2*α*: Eukaryotic translation initiation factor
ERK: Extracellular signal-regulated kinase
GDNF: Glial cell line-derived neurotrophic factor
GI: Gastrointestinal
HO-1: Heme oxygenase 1
HPO-DAEE: 4-Hydroperoxy-2-decenoic acid ethyl ester
HVA: Homovanillic acid
INF-*γ*: Interferon-gamma
iNOS: Inducible nitric oxide synthase
IL: Interleukin
MAPK: Mitogen-activated protein kinase
MDA: Malondialdehyde
MPP: 1-Methyl-4-phenylpyridinium
MMPs: Matrix metalloproteinases
MRJPs: Major royal jelly proteins
MPTP: 1-Methyl-4-phenyl-1,2,3,6-tetrahydropyridine
NF-*κ*B: Nuclear factor kappa-B
NGF: Nerve growth factor
NQO1: NAD(P)H quinone dehydrogenase 1
NRF2: Nuclear factor erythroid 2
PB: Pinocembrin
p.o.: Oral gavage
PD: Parkinson's disease
pRJ: Protease-treated RJ
RCT: Randomized control trial
RMNs: Rostral mesencephalic neurons
RJ: Royal jelly
ROS: Reactive oxygen species
S100B: Calcium-binding protein B
SNC: Substantia nigra pars compacta
SOD: Super oxide dismutase
TH: Tyrosine hydroxylase
TNF: Tumor necrosis factor
TAR: Total antioxidant reactivity
TRAP: Total reactive antioxidant potential.
